# Trace metal concentrations in *Posidonia oceanica *of North Corsica (northwestern Mediterranean Sea): use as a biological monitor?

**DOI:** 10.1186/1472-6785-6-12

**Published:** 2006-09-11

**Authors:** Marc Gosselin, Jean-Marie Bouquegneau, Frédéric Lefèbvre, Gilles Lepoint, Gerard Pergent, Christine Pergent-Martini, Sylvie Gobert

**Affiliations:** 1 Mare center, Oceanology, University of Liège, Sart-Tilman, B6, 4000 Liège, Belgium; 2 Service Public Fédéral; Direction Générale Environnement, Service Maîtrise des Risques, Eurostation – Bloc II, Place Victor Horta 40, boite 10, 1060 Bruxelles, Belgium; 3 Equipe Ecosystèmes Littoraux, Faculty of Sciences, University of Corsica 20250 Corte, France

## Abstract

**Background:**

Within semi-closed areas like the Mediterranean Sea, anthropic wastes tend to concentrate in the environment. Metals, in particular, are known to persist in the environment and can affect human health due to accumulation in the food chain. The seagrass *Posidonia oceanica*, widely found in Mediterranean coastal waters, has been chosen as a "sentinel" to quantify the distribution of such pollutants within the marine environment. Using a technique similar to dendrochronology in trees, it can act as an indicator of pollutant levels over a timeframe of several months to years. In the present study, we measured and compared the levels of eight trace metals (Cr, Ni, Cu, Zn, As, Se, Cd, and Pb) in sheaths dated by lepidochronology and in leaves of shoots sampled from *P. oceanica *meadows collected from six offshore sites in northern Corsica between 1988 and 2004; in the aim to determine 1) the spatial and 2) temporal variations of these metals in these areas and 3) to compared these two types of tissues.

**Results:**

We found low trace metal concentrations with no increase over the last decade, confirming the potential use of Corsican seagrass beds as reference sites for the Mediterranean Sea. Temporal trends of trace metal concentrations in sheaths were not significant for Cr, Ni, Cu, As or Se, but Zn, Cd, and Pb levels decreased, probably due to the reduced anthropic use of these metals. Similar temporal trends between Cu levels in leaves (living tissue) and in sheaths (dead tissue) demonstrated that lepidochronology linked with Cu monitoring is effective for surveying the temporal variability of this metal.

**Conclusion:**

Leaves of *P. oceanica *can give an indication of the metal concentration in the environment over a short time period (months) with good accuracy. On the contrary, sheaths, which gave an indication of changes over long time periods (decades), seem to be less sensitive to variations in the metal concentration in the environment. Changes in human consumption of metals (*e.g*., the reduction of Pb in fuel) are clearly reflected in both organs. These results confirm that *P. oceanica *is a good bioindicator of metals and a good biomonitor species for assessing Cu in the environment.

## Background

The Mediterranean Sea is one of the most nutrient-depleted seas and is a characteristic oligotrophic system; however, trace metal concentrations are generally high [1], particulary in its surface waters [2]. The Mediterranean coastline is among the most intensively utilized in the world. The approximately 70,000 km coastline (including all islands) receives over one hundred million visitors per year from all over the world. As a result, the Mediterranean coast and the coastal waters have experienced an increasing impact of tourism in addition to the effects of resident demographic growth. Other anthropogenic impacts include agriculture and mariculture as well as wild fisheries, industry, and navigation [3].

**Figure 1:**
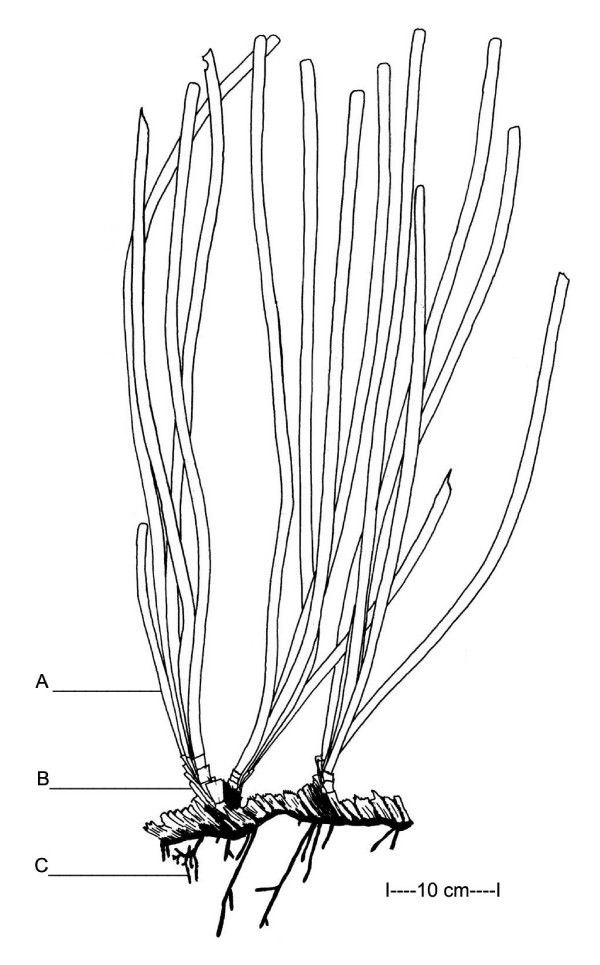
**The Mediterranean seagrass *Posidonia oceanica *(L.) Delile**. Different seagrass organs. A: Leave; B: Dead sheath; C: Root. (Modified from [65]).

The endemic seagrass *Posidonia oceanica *(L.) Delile forms meadows that are crucial to the health and function of coastal ecosystems (Figure 1). Over the last 30 years, frequent alterations and regressions of these meadows have been noted (*e.g*., [4,5]). These alterations are often linked to human activities such as tourism, pollution by urban and industrial centers, and coastal facilities [6-8]. Biological pollution in the Mediterranean Sea [9,10] increases the vulnerability of *P. oceanica *systems [11,12]. Because of the importance of this keystone macrophyte, *P. oceanica *is now considered a threatened species requiring protection and is listed in the 'Habitats Directive' of the European Community [13,14].

*P. oceanica *has been recognized as an effective tool for investigating the coastal environment because it has a widespread distribution around the Mediterranean basin, is fixed on the bottom, and is sensitive to both pollution and human activities. Markert et al. [15] define a "bioindicator" as an organism that contains information about the quality of the environment and a "biomonitor" as an organism that contains quantitative information regarding the quality of the environment. Previous studies suggest that *P. oceanica *can be used as a bioindicator [6,16-18]. In addition, lepidochronology [19-21], phenology [22], and tissue contents [23-26] can be used to examine historical changes in the environment.

**Figure 2:**
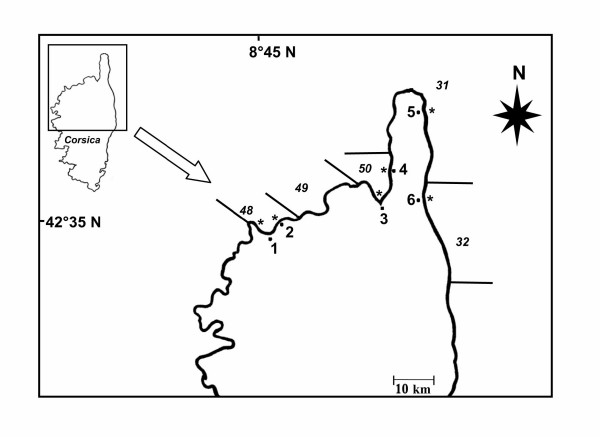
**Location of sampling sites**. *1: Calvi, 2: Lumio, 3 Saint-Florent, 4: Nonza, 5: Macinaggio and 6: Bastia; divided into "Zones homogènes" of the coast according the "Agence de l'eau" (France).

The aim of this work was to 1) verify the efficiency of the magnoliophyta *P. oceanica *as a bioindicator of the baseline of trace metal pollution at a regional scale. We measured eight trace metal contents (Cr, Ni, Cu, Zn, As, Se, Cd, and Pb) in dated remains of sheaths (lepidochronology) at six sites close to urban zones in northern Corsica (Figure 2). 2) attempt to use *P. oceanica *as a biomonitor by comparing, over the last 10 years, trace metal contents measured in leaves with results found in sheaths at Calvi.

## Results and discussion

### *P. oceanica *as bioindicator

#### Shoot and water characteristics in northern Corsica

The November 2001 and February 2002 data show that the column and interstitial waters in northern Corsica were very nutrient poor. In the water column, the maximum values measured in November for the Calvi area were 2.19, 0.13, and 0.09 μM for NH_4_^+^, NO_3_^-^, and orthophosphate, respectively, but, more often, levels were below detection. Sediment pore water concentration ranges were 0.09–3.03, 0.08–7.41, and 0.02–0.59 μM for NH_4_^+^, NO_3_^-^, and orthophosphate, respectively.

In comparison to the northwestern Mediterranean Sea, the waters of the studied areas along the island of Corsica are characterized by low nitrogen and phosphorus levels [1,27]. The hydrological features of this region favor low nutrient availability, specifically, weak terrestrial inputs and irregular deep water shelf-slope exchanges linked to meteorological events [28]. Nutrient inputs are often restricted to the winter to early spring period.

In spite of their very different structural aspects (substrate, patchiness, etc.), the six studied meadows exhibited similar biometric parameters (Tables 1 and 2). All the meadows were dense [29], had a high leaf biomass, and a high leaf surface area. Only plants from Saint-Florent site had leaf biomass and nitrogen contents lower than in the other meadows. This high biomass and shoot densities recorded at all sites in the current study (except for Saint-Florent) indicate that the meadows are dense and healthy [30]. The epiphyte community was particularly developed in Bastia Bay and represented more than one sixth of the leaf biomass. The *P. oceanica *meadow of the Calvi Bay is one of the most productive meadows of the north Mediterranean coast [31] (*i.e*., leaf + rhizome production at a 10-m depth = 760 g_DW _m^-2 ^y^-1^) and shows high shoot biomasses and shoot densities [32].

#### Trace metals in meadows of northern Corsica

The mean concentrations of Cr, Ni, Cu, Zn, As, Se, Cd, and Pb in dead sheaths of *P. oceanica *between 1997 and 2001 varied according to the metals and the sites (Table 3).

The lowest Cr concentrations were measured at the Lumio (22 ± 14 μg.g_DW_^-1^) and Calvi (20 ± 12 μg.g_DW_^-1^) sites, and the highest levels were observed at the Nonza site. The metal concentrations at Calvi and Lumio were significantly lower than at Macinaggio (*P *< 0.001 and *P *< 0.01, respectively) and Bastia (*P *< 0.001 for both). Samples from the Calvi area also had lower metal concentrations than those from Saint-Florent (*P *< 0.05). Cr concentrations at the Nonza site (123 ± 41 μg.g_DW_^-1^) were significantly higher than at all other sites except Bastia (*P *> 0.05; *P *< 0.001 vs. Calvi and Lumio; *P *< 0.01 vs. Saint-Florent; *P *< 0.05 vs. Macinaggio).

The highest Ni concentrations were observed in samples from the Nonza site (111 ± 52 μg.g_DW_^-1^). These values were significantly higher than those measured at all other sites (*P *< 0.001 vs. Calvi and Lumio and *P *< 0.01 vs. Saint-Florent and Macinaggio) except for the Bastia site (*P *> 0.05). Ni concentrations from Calvi (8 ± 6 μg.g_DW_^-1^) and Lumio (9 ± 6 μg.g_DW_^-1^) were significantly lower than at all other sites (*P *< 0.01 and *P *< 0.05 vs. Saint-Florent; *P *< 0.001 and *P *< 0.05 vs. Macinaggio; *P *< 0.001 for both vs. Bastia).

Cr and Ni levels in *P. oceanica *show similar spatial variations around the northern Corsican coast. At Nonza, high concentrations of these two metals were found. Wright and Wellbourn [33] emphasize that mining operations may produce Cr and Ni particles that contaminate ground and surface water in the ecosystem. Thus, the high concentration around the Nonza station could be due to an asbestos quarry that lies 15 km from Canari, where wastes were directly dumped into the sea between 1941 and 1965 [34]. Indeed, shale contains asbestos, which is rich in Cr and Ni. The fact that Calvi and Lumio show lower concentrations than Saint-Florent can be explained by the hydrologic current distribution in Gulf of Saint-Florent and the fact that Calvi and Lumio were not contaminated with asbestos. The increase in Cr and Ni concentrations observed these last years in meadows in front of Bastia could reflected the urban and industrial activity of the city. Because Ni is known to be bioconcentrated [33], particularly by plants, we suggest that a survey of this trace metal should be conducted in the upper trophic levels around Nonza and Bastia.

Cu concentrations in north Corsica *P. oceanica *meadows were consistently low. The lowest concentrations were found at Lumio (9 ± 1 μg.g_DW_^-1^), Macinaggio (9 ± 2 μg.g_DW_^-1^), and Calvi (10 ± 3 μg.g_DW_^-1^). These concentrations were significantly lower than at Saint-Florent, Nonza, and Bastia (*P *< 0.01 for Calvi vs. Saint-Florent and for Calvi vs. Nonza; *P *< 0.001 for all others). The highest Cu values of 16 ± 7 μg.g_DW_^-1 ^and 15 ± 3 μg.g_DW_^-1 ^were observed in samples collected from Saint-Florent and Bastia, respectively.

The high Cu contamination found around Bastia may be related to the use of this metal as a fungicide in surrounding vineyards (Cap Corse exploitations) and as an antifouling agent in boat paints. The Cu level in northern Corsica, however, was lower than along the French or Italian coasts [20,34]. Pasqualini et al. [35] found a correlation between the characteristics of *P. oceanica *meadows and Hg, Cu, Pb, Cd, and As contamination levels. According to their classification scheme, the Cu levels at the Corsican sites can be considered normal or background (<10–14 μg.g_DW_^-1^), except for Saint-Florent and Bastia, which had "subnormal"concentrations (16 ± 7 and 15 ± .3 μg.g_DW_^-1^, respectively). The concentrations in these two areas likely result from local activities in the harbors and vineyards. The impact of antifouling Cu painting, including an increase in Cu levels in oysters or water, has already been described in other marine areas [36,37].

The Zn concentrations were lowest at Lumio (14 ± 3 μg.g_DW_^-1^) and Macinaggio (15 ± 4 μg.g_DW_^-1^). These Zn levels were significantly lower than at Calvi (*P *< 0.001), Saint-Florent (*P *< 0.001), and Bastia (*P *< 0.001 vs. Lumio and *P *< 0.01 vs. Macinaggio). The highest Zn concentration was found at Bastia (31 ± 8 μg.g_DW_^-1^), which was 2-fold higher than at Nonza (16 ± 3 μg.g_DW_^-1^; *P *< 0.001) and significantly higher than at Calvi (20 ± 4 μg.g_DW_^-1^; *P *< 0.05). Also, the Zn concentration at Nonza was lower than at Saint-Florent (23 ± 4 μg.g_DW_^-1^; *P *< 0.01).

The Zn levels were not described by Pasqualini et al. [35], but our results show that the concentrations in *P. oceanica *sheaths from Corsica are low in comparison with those in French meadows [4,20]. The levels of Zn at the Saint-Florent and Nonza sites decreased between 1996 and 2001. Zn is an essential micronutrient for plant growth [33]. This metal may diffuse from the sediment, and it is transported into the intracellular space by passive diffusion [38]. The levels encountered at our study sites are probably representative of a relatively unaffected area [20].

Concentrations of As varied between a minimum at Saint-Florent and Calvi (14 ± 7 and 16 ± 6 μg.g_DW_^-1^, respectively) and a maximum at Macinaggio (21 ± 11 μg.g_DW_^-1^); however, the differences were not statistically significant.

Very few previous study has examined As accumulation in *P. oceanica *leaves and dead sheaths [35]. Our results, which are particularly homogenous, indicate that As levels were higher than those reported for the seagrasses *Zostera capricornii *[39] and *Zostera marina *[40]. In contrast, our results indicate the *P. oceanica *meadows from Corsica have very high levels of As compared to the levels reported previously for the Mediterranean [35]. This metal can enter the aquatic environment through both natural (weathering and volcanic activities) and anthropogenic (combustion of municipal waste and use of herbicides and antifouling agents) activities. We suspect natural inputs, but due to the limited amount of data available, additional studies, including the relationship with sediment characteristics (geology and physicochemical), are necessary to determine the distribution of this metal in Mediterranean seagrasses.

The highest Se concentrations were observed at Nonza (80 ± 15 μg.g_DW_^-1^) and the lowest at Saint-Florent (56 ± 35 μg.g_DW_^-1^); however, no statistically significant differences between sites were found.

Se concentrations were uniform around northern Corsica, and no temporal trend was detected over the last decade. The range of concentrations (13–138 μg.g_DW_^-1^) found in sheaths of Corsican *P. oceanica *were 100-fold higher than reported for other seagrasses [41]. These high concentrations of Se are probably due to a nonanthropogenic effect, such as weathering of rocks rich in Se or upwelling, because Se is concentrated in deep water [42]. Watanabe [43] stressed that Se could enhance the effect of Hg in organisms. Moreover, the Se concentration is known to increase with the trophic level [39]. Because Se is an element that could threaten marine ecosystems, we suggest that future studies investigate its dynamics in seagrasses.

Cd concentrations were low at Bastia (0.4 ± 0.1 μg.g_DW_^-1^), and the highest concentrations were found at Calvi (1.1 ± 0.8 μg.g_DW_^-1^) and Nonza (1.2 ± 0.4 μg.g_DW_^-1^). The Cd levels at Calvi were significantly higher than at Lumio (*P *< 0.001), Saint-Florent (*P *< 0.01), Macinaggio (*P *< 0.001), and Bastia (*P *< 0.001). The concentrations at the Nonza site were higher than at Lumio (*P *< 0.001), Saint-Florent (*P *< 0.05), Macinaggio (*P *< 0.001), and Bastia (*P *< 0.001).

The Cd concentrations at the Nonza and Calvi sites in *P. oceanica *sheaths were higher than at other Corsican sites (Table 3) and even other Mediterranean sites [20,44] (Table 4). In 1987, Mingelbier (unpublished data) found 2-fold more Cd in *P. oceanica *leaves in Calvi than in Bastia. The Bay of Calvi is located near the protected area of the Scandola and is considered an uncontaminated site [20,45]. The enrichment of Cd in sheaths is not clearly linked with anthropogenic activities, but a natural phenomenon such as an upwelling of deep waters rich in Cd has been suggested for this region [20,46]. A similar system may explain the behavior observed at the Nonza site.

Pb concentrations were a homogenous 7 μg.g_DW_^-1 ^except at the Bastia site, which had a higher mean concentration (25 ± 29 μg.g_DW_^-1^). The Pb levels at the Bastia site were significantly higher than at Saint-Florent (P < 0.01) and the other sites (*P *< 0.001).

Like Port-Cros [6], the Bay of Calvi is considered to be an area of low Pb contamination. Except for the Bastia site, the Corsican sites investigated here had Pb levels similar to Calvi and can be classified as sites with low Pb contamination. Our results from Calvi, Lumio, Saint-Florent, Nonza, and Macinaggio are similar to those reported for unpolluted areas, such as Liscia Bay in Sardinia [44] and the Lavezzi Natural Park in Corsica [20]. The Pb contamination in the vicinity of Bastia (50,000 inhabitants) is extreme according the classification of Pasqualini et al. [35] and may be related to local shipping and industrial activities.

Although our study was conducted at very unpolluted sites, it showed that when using bioindicators such as *P. oceanica *sheaths and the notion of a background level, local conditions such as geochemistry and hydrodynamic parameters must be taken into consideration. As a result, it can be difficult to distinguish the source of contamination using bioindicators alone.

### *P. oceanica *as biomonitor

In this part of the study, we present the metal concentration history in sheaths sampled from six meadows and in leaves sampled from Calvi. Then we compared the metal concentration history in leaves and sheaths sampled from Calvi (Table 5). Leaf samples (living tissues) were collected in 1993, 1994, 2003, and 2004, and sheaths (dead tissues) were sampled in 2001 and 2002. This sampling design allowed us to retrospectively evaluate a 10-year period from 1992 to 2002. It was therefore possible to compare the two types of organs to determine if sheaths conserve a precise record of the metal concentrations in the leaves.

#### Temporal evolution of metal concentrations in dead sheaths

**Figure 3:**
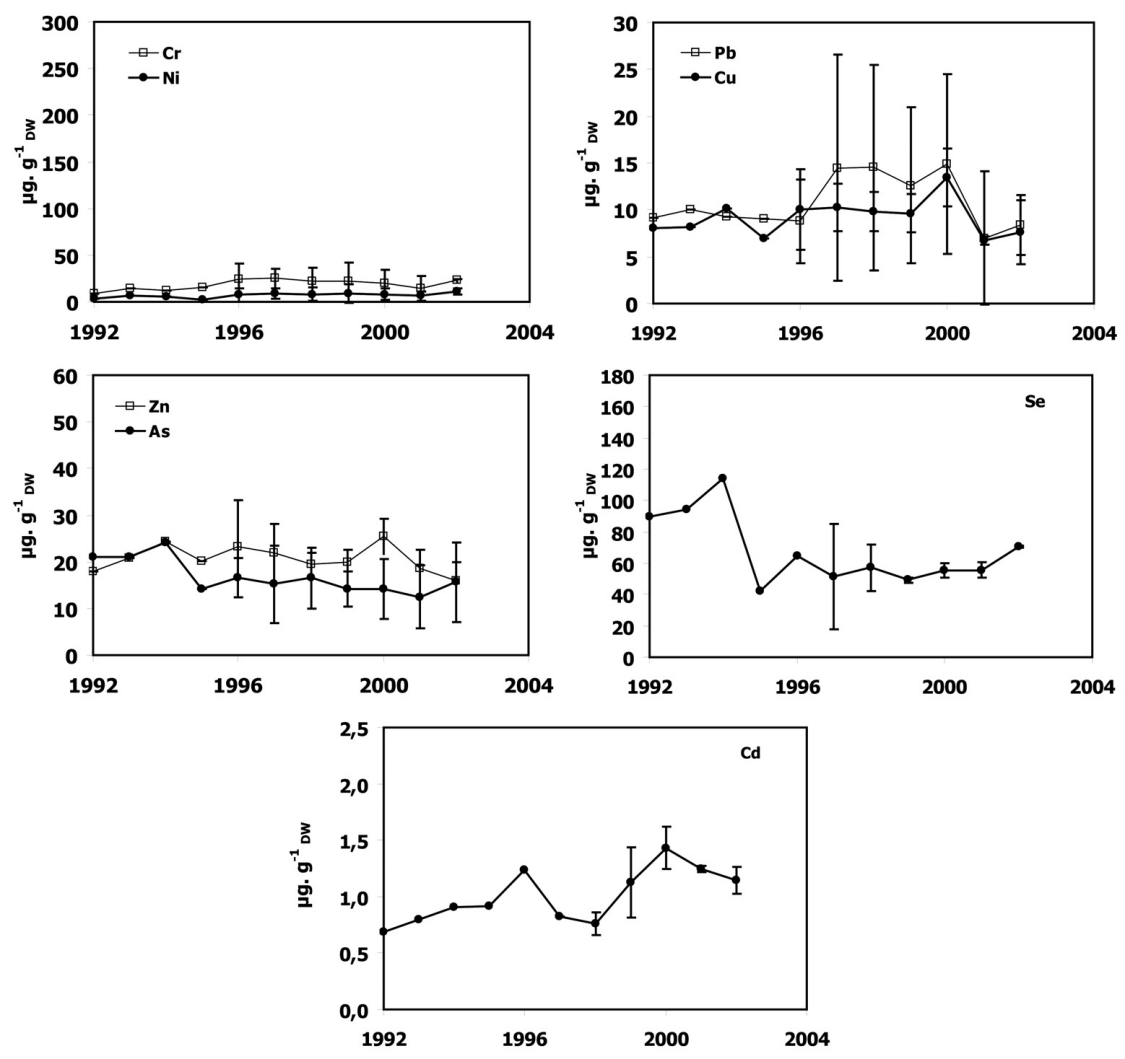
**Metal concentrations in *P. oceanica *dead sheaths sampled in the Bay of Calvi**. Cr, Ni, Cu, Zn, As, Se, Cd and Pb concentrations as a function of lepidochronological years were expressed in μg g_DW_^-1 ^(mean ± standard deviation).

**Figure 4:**
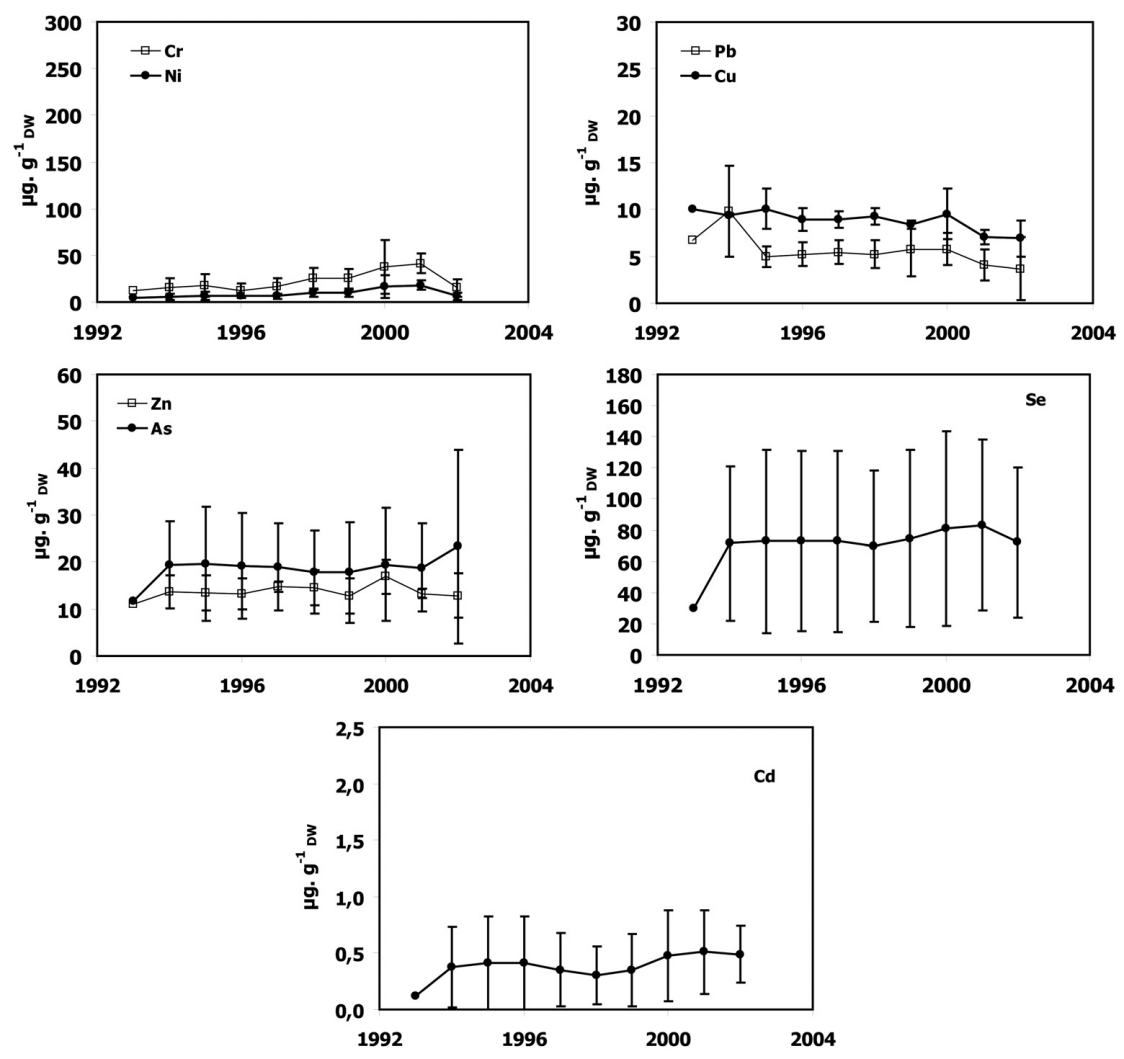
**Metal concentrations in *P. oceanica *dead sheaths sampled in front of Lumio**. Cr, Ni, Cu, Zn, As, Se, Cd and Pb concentrations as a function of lepidochronological years were expressed in μg g_DW_^-1 ^(mean ± standard deviation).

**Figure 5:**
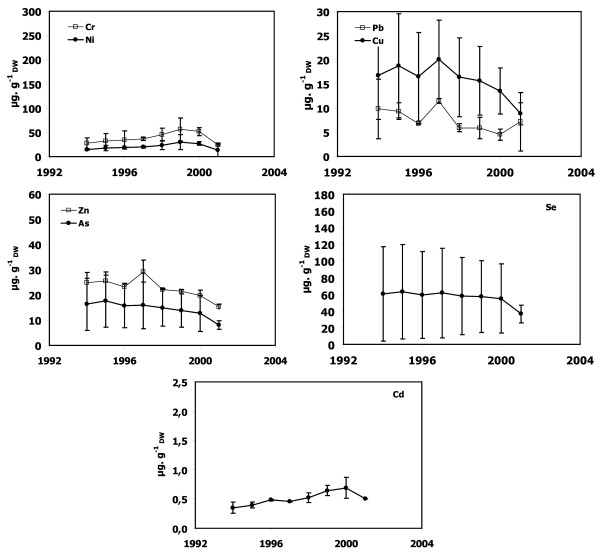
**Metal concentrations in *P. oceanica *dead sheaths sampled in front of Saint-Florent**. Cr, Ni, Cu, Zn, As, Se, Cd and Pb concentrations as a function of lepidochronological years were expressed in μg g_DW_^-1 ^(mean ± standard deviation).

**Figure 6:**
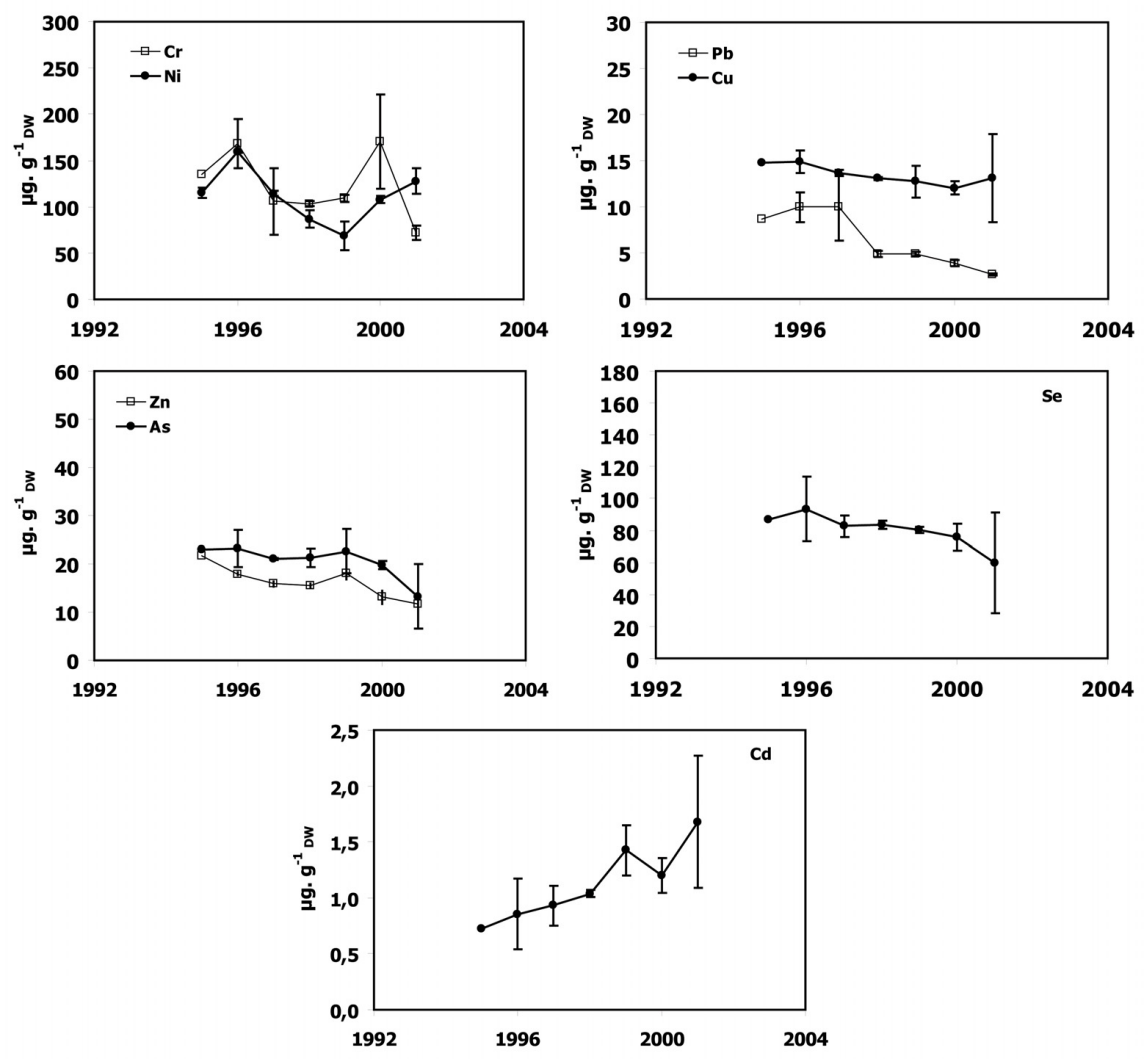
**Metal concentrations in *P. oceanica *dead sheaths sampled in front of Nonza**. Cr, Ni, Cu, Zn, As, Se, Cd and Pb concentrations as a function of lepidochronological years were expressed in μg g_DW_^-1 ^(mean ± standard deviation).

**Figure 7:**
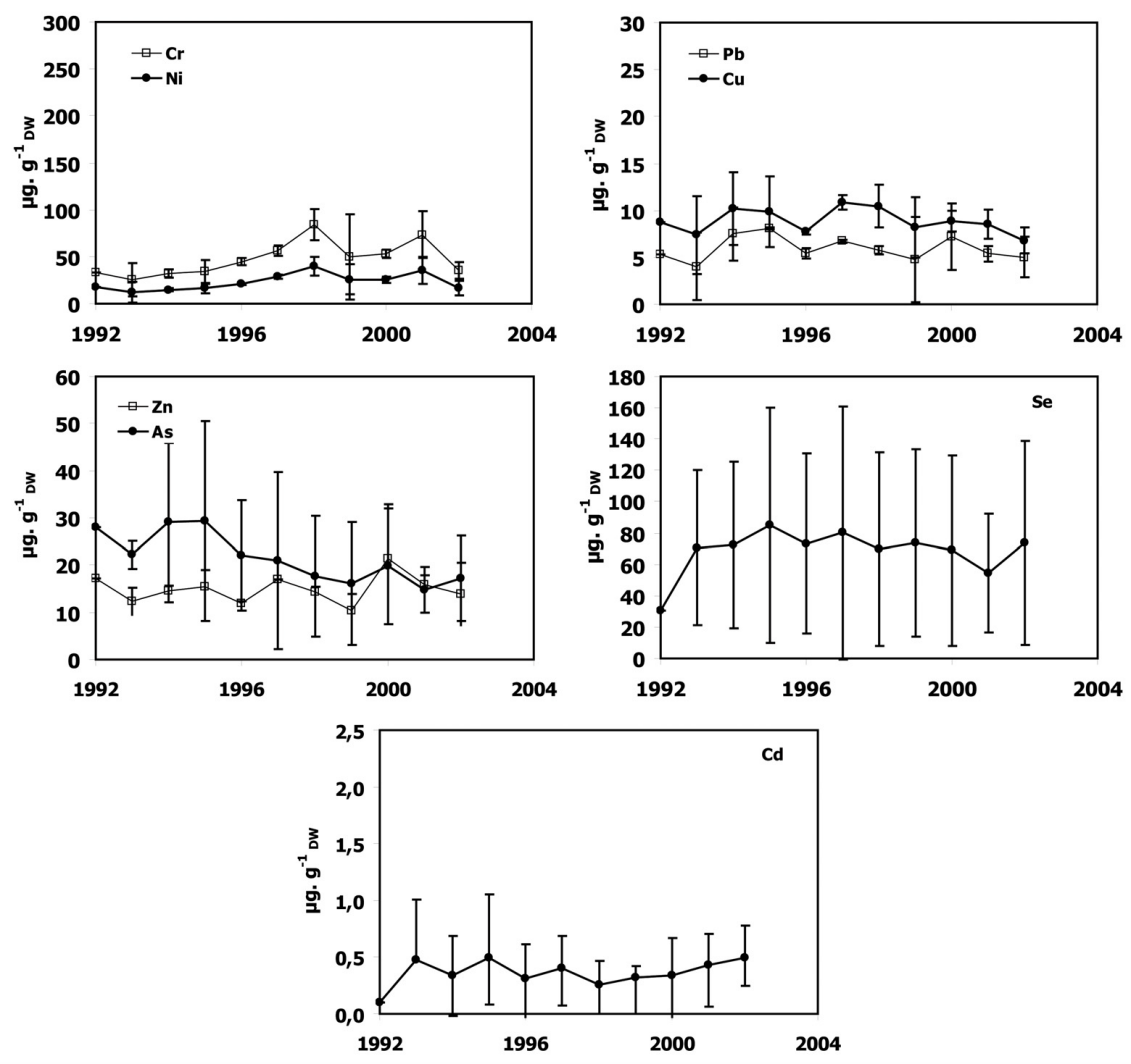
**Metal concentrations in *P. oceanica *dead sheaths sampled in front of Macinaggio**. Cr, Ni, Cu, Zn, As, Se, Cd and Pb concentrations as a function of lepidochronological years were expressed in μg g_DW_^-1 ^(mean ± standard deviation).

**Figure 8:**
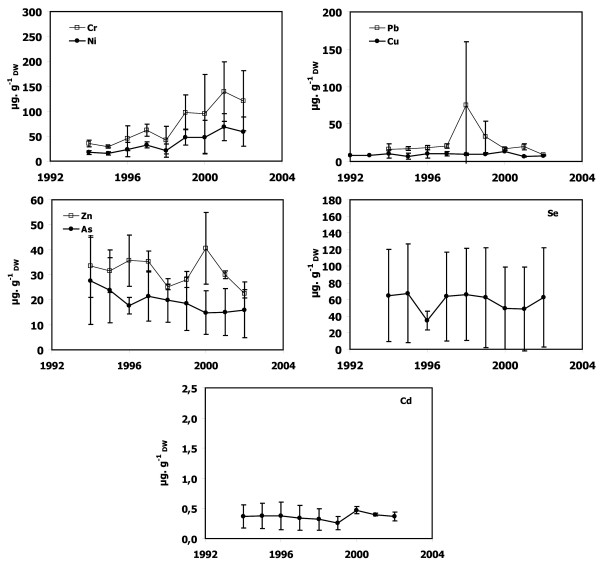
**Metal concentrations in *P. oceanica *dead sheaths sampled in front of Bastia**. Cr, Ni, Cu, Zn, As, Se, Cd and Pb concentrations as a function of lepidochronological years were expressed in μg g_DW_^-1 ^(mean ± standard deviation).

Temporal trends of Cr, Ni, Cu, Zn, As, Se, Cd, and Pb concentrations in dead *P. oceanica *sheaths from lepidochronological years 1992–1995 and 2002 are shown for samples from Calvi (Figure 3), Lumio (Figure 4), Saint-Florent (Figure 5), Nonza (Figure 6), Macinaggio (Figure 7), and Bastia (Figure 8). In some cases, it was not possible to obtain results as far as back as 1992.

Dead sheath samples from Calvi, Lumio and Macinaggio exhibited no significant temporal trend for any trace metals (Figures 3, 4 and 7). In contrast, there were obvious trends in the dead sheath data from the other sites. At Saint-Florent, the Zn level decreased significantly between 1994 and 2001 (*P *< 0.05; Figure 5). At Nonza (Figure 6), the Zn level decreased significantly between 1996 and 2001 (*P *< 0.05), with a slight increase in 1999 (18 ± 1 μg.g_DW_^-1^) and the Cr concentrations are lower in 2001 than in 1996 and 2000 (*P *< 0.05). Additionally, between the years 1996/1997 and 2000/2001, the Pb concentrations at Nonza decreased significantly (*P *< 0.05). In contrast Cd levels increase significantly between the years 1996 and 2001 (*P *< 0.05). At Bastia (Figures 8), a significant increase in Cr and Ni concentrations were observed between the years previous and following 1998/1999.

#### Temporal evolution of metal concentrations in living leaves

**Figure 9:**
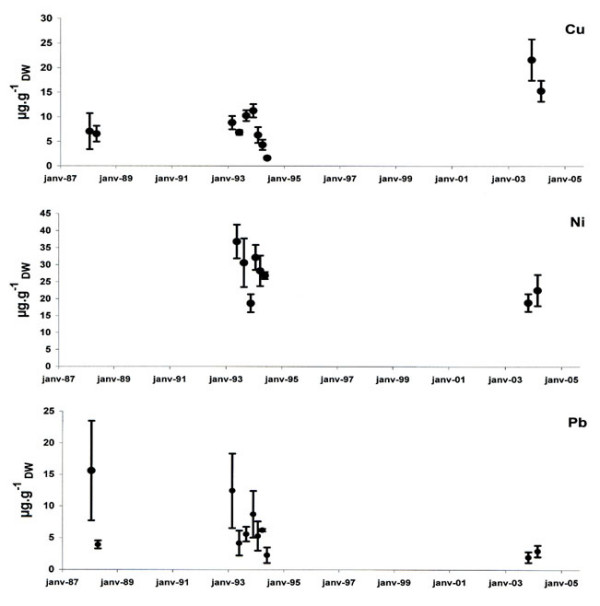
**Metal concentrations in leaves between 1988 and 2004 in the Calvi area**. Cu, Ni and Pb concentrations were expressed in μg g_DW_^-1 ^(mean ± standard deviation).

**Figure 10:**
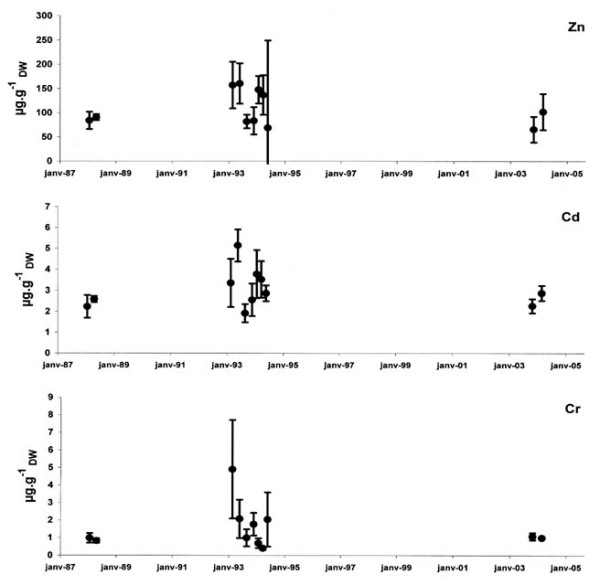
**Metal concentrations in leaves between 1988 and 2004 in the Calvi area**. Zn, Cd and Cr concentrations were expressed in μg g_DW_^-1 ^(mean ± standard deviation).

The concentrations in living leaves differed according to the trace elements and generally could be ranked from highest to lowest as follows: Zn > Ni > Cu > Pb > Cd > Cr (Tables 5 and 6). Figures 9 and 10 show the temporal variations of Cr, Ni, Cu, Zn, Cd, and Pb in leaves of *P. oceanica *collected between 1988 and 2004.

*P. oceanica *had higher levels of Cu in leaves in 2003 and 2004 than in the preceding years. In contrast, the Pb concentrations decreased significantly over the last 15 years (Table 6). Leaves present a slight but significant decrease in Zn concentrations between the years 1994 and 2003 (HSD Tukey, P = 0.049). Due to heterogeneity, the other trace metals measured (Cr, Ni, Zn, and Cd) did not show significant temporal variations between 1988 and 2004 (Table 6).

The stable Cu concentration through time in sheaths from north Corsica contrasts with results obtained in *P. oceanica *sheaths from Sardinia [44]. In Sardinia, which is considered unpolluted, an increase of Cu concentration was found between 1978 and 1994 in the dead sheaths. Cu is an essential element for plant growth and photosynthesis, and these phanerogams probably have the capacity to regulate this metal [23,26]. These authors showed that the Cu levels in sheaths compare well with the environmental levels [44]. The factors that influence the temporal variation in sheaths remain unknown. Conflicting results for the temporal history between leaves and sheaths make it difficult to generalize, but there appears to be a tendency for high Cu absorption by old and dead macrophyte tissues [47].

Using dead sheaths, we observed a decrease in the concentration of Cd in *P. oceanica *from northern Corsica over the last 15 years. This tendency was confirmed by comparing values measured in sheaths from 1992 and 2002 with those reported by Pergent-Martini [48] between 1970 and 1992. Previous studies showed that Cd tissue concentrations in seagrasses reflected the Cd in the environment waters [49] or in the sediment [50]. Thus, the observed decrease suggests a temporal decrease of Cd concentrations in seawater. Indeed, in the Bay of Calvi, recent observations have shown long-term changes (1979–1998) in climatic and environmental conditions [27]. These changes have affected the vertical stability of the water column and reduced nutrient loading by reducing 1) rainwater inputs (reduction of soil leaching) and 2) winds that initiate upwelling. These changes could generate a reduction in the natural input of Cd into the bay. Further studies, however, are needed to test this hypothesis.

At Calvi, the Pb concentrations in leaves decreased between 1986 and 2004 and those in sheaths decreased between 1970 and 2002. Moreover, the same trend was observed in sheaths between 1997 and 2001 at the Nonza site. This trend agrees with the decreasing Pb concentration in the atmosphere [51] and surface seawater [52], and it is likely the result of the progressive reduction in the use of Pb additives in automobile fuel as suggested by Roméo et al. [20], who also found a progressive decrease in Pb levels in dead sheaths of *P. oceanica*. Long-term climate change for Cd, which limits the entry of elements in upper layers of the sea, could also play a role in the decreasing trend in Pb levels. Conversely, temporal trends could be masked by modifications (aging) of the sheaths with time because Pb can bind the outer surface of the plant [53]. The tissues of old sheaths tend to become porous, which increases the available surface for passive adsorption [48].

Locally, we observed a decrease in several metals over recent years in *P. oceanica *sheaths, namely in Zn at Nonza and Saint-Florent, Cd at Calvi, and Pb at Calvi and Nonza. Similarly, Cappelli et al. reported that [54], in fish and decapoda in the Ligurian Sea (northwestern Mediterranean), the levels of Pb, Mn, Cu, Zn, Cd, and Hg but not Se decreased. Thus, in contrast to what is commonly reported for the Mediterranean Sea, none of the metals studied here seem to have increased along northern Corsican coast during the past 15 years.

The marine coastal environment is subject to the entry of trace metals from several sources, including both natural (run-off, atmospheric, deposition, etc.) and anthropogenic (industry, urban, etc.) processes. Furthermore, the bioavailability of trace metals is affected by sediment, pH, and redox potential [55], and the environmental conditions influence the accumulation processes of living sheaths as well as the degradation processes of dead sheaths. Therefore, the accumulation of trace metals in *P. o*ceanica depends on 1) the metal element, 2) the organ (live blade, live sheath, or dead sheath), and 3) the study site (Table 7).

#### Temporal history of trace metal concentrations recorded in dead sheaths and living leaves of P. oceanica from the Calvi area

Comparison of the dead and living tissue of *P. oceanica *shoots at Calvi showed that the concentrations were significantly different (Table 5). Cr and Pb concentrations were significantly higher in sheaths than in leaves, whereas concentrations of Ni, Cd, and Zn were higher in leaves than in sheaths.

These different concentrations could be the result of 1) methodological factors, in that we compared dead sheaths and living leaves, even though living leaves are equivalent to living sheaths plus living blades; and 2) physicochemical factors, in that during decay, dead sheaths desorb Ni, Cd, and Zn and adsorb Cr and Pb. Our results do not support the conclusions of previous studies [16] that compared metal concentrations in living leaves and dead sheaths. For *P. oceanica*, the previous studies found that Cr, Ni, Cu, Zn, Cd, and Pb are present at higher concentrations in living leaves (aboveground compartment) than in dead sheaths. The difference can be explained by the fact that our study focused on the sheath degradation process rather than the leaf accumulation process.

Moreover, in other data from the Adriatic Sea, Kljaković-Gaśpić et al. [56] showed that the oldest sheaths and the dead sheaths of *P. oceanica *exhibited similar Cd and Pb concentrations. This difference could be due to the process used to measure metals in the living tissues. Our results correspond to a mean concentration measured from one entire shoot, and it has been demonstrated that leaf aging causes a dilution effect [57]. Thus, the extent of the change in concentration can be minimized (Ni, Zn, and Cd) or maximized (Cr and Pb) by the variation in the metal concentration, which increases with leaf age.

We suggest that future studies should 1) rigorously examine the metal concentrations along a leaf age gradient from the same shoot, and 2) compare trace metal concentrations in dead sheaths with measures in living sheaths and not in the whole living leaves. Although many of the metals did not follow the expected patterns, our results suggest that Cu concentrations were the same in both living leaves and dead sheaths. Therefore, data on Cu for dead sheaths could be used to reconstruct the original leaf concentrations. Gagnon [58] noted that mean Cu concentrations in plants were between 5 and 20 μg.g_DW_^-1^, which correspond with data found in *P. oceanica *leaves and sheaths (table 3, 5 and 6) and confirm the good health status of north Corsica meadows.

## Conclusion

In the Mediterranean Sea, organic and trace metal pollution has increased over the last 20 years [59,60], and this has had a perceptible effect on marine environments [61]; however, the seagrass meadows investigated in this study can be described as healthy in terms of density and biomass. The nutrient levels in the surrounding waters were low, and the trace metal levels in dead sheaths of *P. oceanica *were, in most cases, comparable to meadows in unpolluted areas. Thus, the meadows investigated in this study appear to be relatively unaffected by the increase of industrial, agricultural, and urban activities, and the surface waters exhibit low anthropogenic (organic and trace metals) discharges. Seagrasses in northern Corsica are currently safe from human pollution, although there are some sites showing impacts of urban centers (*i.e*., Bastia) or mining waste (*i.e*., Nonza).

Our study shows that *P. oceanica *can be used as a bioindicator of trace metals in the Mediterranean Sea because of its widespread distribution and because it allows retro dating and smoothing of seasonal variations (lepidochronology). In particular, this plant seems to be useful as a biomonitor for Cu, which is essential for plant growth and metabolism, because variations in its distribution in sheaths reflect the original leaf concentrations. The sheaths were less useful for recording changes in the concentrations of Cr, Ni, Zn, Cd, and Pb. Unfortunately, the As and Se levels could not be tested because they had only been measured in sheaths and not in leaves. Further studies on these latter eight metals should allow determination of whether they can be examined using lepidochronology techniques.

## Methods

### Study sites

We sampled six meadows just offshore from the cities of Calvi, Lumio, Saint-Florent, Nonza, Macinaggio, and Bastia along the northeastern and northwestern Corsican coasts (Table 8). The west coast, or "old Corsica", is dominated by granite rock, whereas the northeast coast, or "alpine Corsica", is dominated by shale. The six investigated meadows have different environmental characteristics that encompass the range of characteristics found in the Mediterranean Sea (Figure 2). Off the shore of Calvi, at a depth of 10 m, the meadow is dense and continuous and colonizes a sandy substratum with a 2% slope [62]. The meadow at Saint-Florent is patchy with rocks between the stands of *P. oceanica*. At Nonza, the meadow colonizes a pebbly substrate. The coasts of south Saint-Florent have a slight slope of sand or rock, whereas the slope is steeper and rocky around Nonza. These two meadows are well separated. At Macinaggio and Bastia, the coast is principally rock, and the meadow grows on a sandy plain that is damaged by trawling [34].

The French coasts have been divided into 50 zones ("zones homogènes") by the Agence de l'Eau. Each zone is characterized according to physical criteria (type of coast, currents, etc.), the quality and biodiversity of the ecosystems, human activities (fisheries, fish farming, urbanization, etc.) and anthropogenic input (sewage, dredged materials, etc.) (Table 1).

### Sampling for trace metal measurements in living tissues

Ten orthotropic shoots were collected at the Calvi site from February 1988 to December 2004 next to the STARESO station (February and May 1988; March, June, September, and December 1993; February, April, and June 1994; November 2003; and December 2004). In the laboratory, epiphytes were removed with a shard of glass to avoid metal contamination. Leaves from samples collected between 1988 and 1994 were lyophilized and digested using a double boiler, whereas samples collected in 2003 and 2004 were subjected to microwave digestion. For the samples collected between 1988 and 1994, the levels of Cr, Ni, Cu, Zn, Cd, and Pb were measured with an inductively coupled plasma-atomic emission spectrometer (ARL-3510). Recovery ranges with certified QUASIMEME materials (QTM041BT, QTM042BT) were 88 ± 1.5 % to 99 ± 1% for these metals. Samples collected from 2003 and 2004 were analyzed by a certified laboratory (Institut Malvoz-Laboratoire Santé et Cadre de Vie, Liege, Belgium) with an inductively coupled plasma-mass spectrometer (Elan DCR II). A set of certified Material samples (DORM-2, National Research Council, Institute for National Measurement Standards, Ont; Canada) spiked with grade concentration of metals was analyzed to ensure the accuracy of metals. The metal concentrations were expressed as μg.g_DW_^-1^. The change of measuring apparatus is due to a breakdown of the apparatus which we use in routine (ICP AES ARL-3510). All the analyses were carried out by our technician and all the procedure of preparation of the samples was carried out in our laboratory. Rigorous protocols have been followed and intercalibrations have been made. For example, for the Zn concentrations, tests have been realized in the Calvi leaves (2003 and 2004); a correlation test of Spearman rank has been made which show a correlation coefficient of 0,997 at a Pvalue of 0,001. In spite of this breakdown which obliged us has to use another apparatus, our results are comparable and can be regarded as resulting from only one and single series.

### Sampling for trace metal measurements in dead sheaths

At each site during November 2001 and February 2002, density estimates (n = 10) were made at a 10-m depth by a diver [63]. Fifteen shoots from *P. oceanica *plants were collected to measure biometric parameters, and 15 orthotropic rhizomes were collected at each site for measurement the trace metal levels.

Using lepidochronology, it is possible to use the dead sheaths from *P*. *oceanica *to examine the history of metal concentrations in the environment over several decades (Figure 1). The sheath is the basal part of the leaf that remains attached to the rhizome after abscission of the leave apex (blade). The technique of lepidochronology, which is analogous to dendrochronology, is derived from the life cycle of the phanerogam sheaths, which have an annual periodicity with a maximum and a minimum thickness. Cyclical patterns, therefore, allow each sheath to be assigned a chronological date. In this way, it is possible to measure metal concentrations in each sheath and recreate the temporal history of metal concentrations in the environment [6]. Throughout this work, when we use the word "sheath" alone, it refers to a dead sheath.

#### Biometry

In the laboratory, shoots were dissected as follows: the leaves were separated and measured, scraped to remove epiphytes [64], and the biomass (g_DW _shoot^-1^) was measured after lyophilization. The leaf area (cm^2 ^of leaf area shoot^-1^) and leaf area index (m^2 ^of leaves per m^2 ^of substrate) were calculated according to Giraud [30].

#### Water samples

Sediment pore water and water column samples were collected with a syringe for analysis of nutrients (NH_4_^+^, NO_3_^-^, and PO_4_^-^) [62]. Nutrient concentrations were determined using a SAN SKALAR autoanalyzer. The results are expressed in μM, and the detection limit was 0.01 μM for the three nutrients.

#### Dead sheaths

The orthotropic rhizomes were dissected for lepidochronological analysis [19]. The sheaths of three shoots were pooled according to lepidochronological year, pulverized, and lyophilized. A portion of the sample (155 mg) was digested with a mixture of 1 ml of concentrated HNO_3_, 0.1 ml of H_2_O_2_, and 0.25 ml of deionized distilled H_2_O. The mixture was placed in a Teflon bomb and microwaved for 5 min at 300 W, 30 s at 600 W, and 4 min at 250 W. Concentrations of Cr, Ni, Cu, Zn, As, Se, Cd, and Pb were measured in sheaths using an inductively coupled plasma-mass spectrometer and are expressed in μg.g_DW_^-1^.

### Statistical analysis

The trace metal concentrations in living tissues from the Calvi site samples were compared using Repeated Measures Analysis of Variance to determine the significance of differences between the means for different samples. When a difference was detected, a Tukey's HSD post hoc comparison was used to determine whether the difference in mean values was significant. Normality and homoscedasticity were tested using the Kolmogorov-Smirnof test and Bartlett's test, respectively. Data were log-transformed when necessary.

Different tests were used for the trace metal concentrations in dated sheaths from the northern part of Corsica. To test temporal evolution, Repeated Measures Analysis of Variance was used. When a significant difference between samples was detected, Tukey's HSD post hoc comparison was used. Normality and homoscedasticity were tested with the Kolmogorov-Smirnof test and Bartlett's test, respectively. Data was log-transformed when necessary. To test the significance of differences of trace metals between sites, the Kruskall-Wallis test followed by multiple comparisons based on the Kruskall-Wallis rank sums was used.

To compare relative metal concentrations between dated sheaths and living leaves at Calvi, a Mann-Whitney U Test was used. The results were considered statistically significant at *P *≤ 0.01.

## Authors' contributions

MG: analyzed and interpreted the data, and wrote the manuscript.

JMB and GL: contributed to the critical review of the draft.

FL: participated in the in situ work and measurements.

GP and CPM: participated in the in situ work and measurements, and contributed to the critical review of the draft.

SG: designed the study, contributed to the critical review of the draft, and participated in the in situ work and measurements.

All authors read and approved the final manuscript.

## Figures and Tables

**Table 1:** Site characteristics from the "zones homogènes" subdivisions.

**Sites**	**N° "Zones Homogènes"**	**Morphologic characteristics**	**Ecologic level**	**Anthropogenic pressure**
Calvi	48	Open zone Rocky and sandy coasts	Rich	Medium (Tourism)
Lumio	48	Open zone Rocky and sandy coasts	Rich	Medium (Tourism)
Saint-Florent	50	Semi-closed zone Rocky and sandy coasts	Rich	Low (Agriculture, Tourism)
Nonza	50	Semi-closed zone Rocky and sandy coasts	Rich	Low (Agriculture, Tourism)
Macinaggio	31	Open zone Rocky and sandy coasts	Medium	Medium (Tourism)
Bastia	32	Open zone Sandy coasts	Medium	High (Agriculture, Tourism, Industries)

**Table 2:** Characteristics of the six meadows around north Corsican coasts.

	**Density**	**Leaf biomass**	**Epiphyte biomass**	**Leaf Area Index**
	
*Nov 2001*				
Calvi	461 ± 183	451 ± 150	17 ± 21	8.3 ± 2.5
St Florent	433 ± 205	251 ± 71	4 ± 4	4.6 ± 1.3
Nonza	635 ± 179	540 ± 275	3 ± 3	10.3 ± 5.2
*Feb 2002*				
Calvi	554 ± 123	748 ± 155	43 ± 13	11.6 ± 2.4
Lumio	448 ± 186	624 ± 317	18 ± 13	13.0 ± 3
Macinaggio	627 ± 204	765 ± 169	88 ± 29	15.7 ± 3.9
Bastia	509 ± 159	636 ± 160	117 ± 123	12.5 ± 2.6

Density, mean leaf and epiphyte biomasses (g_DW_.m^-2^), leaf area index (m^2^.m^-2^), at the sampling sites in November 2001 (Calvi, St Florent, Nonza) and February 2002 (Calvi, Lumio, Macinaggio and Bastia).

**Table 3:** Metal concentrations (μg g_DW_^-1^) for the period 1997–2001 in sheaths of *P.oceanica*

	**Cr**	**Ni**	**Cu**	**Zn**	**As**	**Se**	**Cd**	**Pb**
**Calvi**	25 ± 18	11 ± 9	9 ± 3	21 ± 4	14 ± 6	52 ± 30	1.1 ± 0.8	11 ± 6
N = 41	(6 – 89)	(2 – 44)	(4 – 17)	(13 – 30)	(6 – 26)	(17 – 115)	(0.6 – 5.6)	(2 – 30)
**Lumio**	22 ± 14	9 ± 6	9 ± 1	14 ± 3	19 ± 9	72 ± 40	0.4 ± 0.3	6 ± 2
N = 19	(6 – 58)	(2 – 25)	(6 – 12)	(10 – 20)	(9 – 38)	(30 – 125)	(0.1 – 0.8)	(1 – 13)
**St Florent**	39 ± 15	21 ± 7	16 ± 7	23 ± 4	14 ± 7	56 ± 35	0.7 ± 0.8	8 ± 3
N = 16	(20 – 73)	(10 – 37)	(7 – 26)	(15 – 32)	(7 – 25)	(20 – 103)	(0.3 – 3.7)	(3 – 14)
**Nonza**	123 ± 41	111 ± 52	13 ± 2	16 ± 3	20 ± 4	80 ± 15	1.2 ± 0.4	6 ± 3
N = 13	(67 – 207)	(46 – 223)	(10 – 16)	(11 – 22)	(9 – 26)	(37 – 108)	(0.6 – 2.1)	(3 – 13)
**Macinaggio**	48 ± 22	23 ± 11	9 ± 2	15 ± 4	21 ± 11	70 ± 45	0.4 ± 0.3	6 ± 2
N = 21	(13 – 96)	(4 – 47)	(4 – 13)	(8 – 30)	(7 – 44)	(23 – 138)	(0.1 – 0.9)	(1 – 10)
**Bastia**	74 ± 50	37 ± 24	15 ± 3	31 ± 8	19 ± 9	58 ± 40	0.4 ± 0.1	25 ± 29
N = 18	(23 – 182)	(11 – 87)	(11 – 21)	(21 – 51)	(8 – 40)	(13 – 109)	(0.2 – 0.5)	(8 – 135)

Metal concentrations sampled in Calvi, St Florent, Nonza, Lumio, Macinaggio and Bastia. (Mean ± Standard deviation, minimum – maximum).

**Table 4:** Comparison of metal concentrations in dead sheaths of *P. oceanica *in the Western Mediterranean Sea.

	**Cr**	**Cu**	**Zn**	**As**	**Cd**	**Pb**	**Reference**
**Pointe Chèvres (France)**	2.8 ± 0.4						Pergent-Martini, 1994
**5 sites Haute Corse (France)**		15.6 ± 6.7 (10–27)		1.4 ± 0.4 (1–1.84)		2.0 ± 1.0 (0.8–3.6)	Camuglio, 1995
**Lavezzi Corse du Sud (France) 1976–1992**		8 ± 2 (6–15)	19 ± 4 (13–30)		1.2 ± 0.2 (0.7–1.5)	5.0 ± 2.9 (1.7–12.0)	Roméo *et al*., 1995
**La Darse (France) 1983–1992**		34 ± 7 (27–46)	55 ± 7 (34–149)		0.5 ± 0.2 (0.3–0.9)	10.4 ± 7.1 (1.6–26.1)	Roméo *et al*., 1995
**Cap Martin (France) 1986–1992**		17 ± 2 (11–36)	61 ± 7 (35–59)		0.8 ± 0.1 (0.6–1.4)	10.0 ± 1.0 (2.5–22.1)	Roméo *et al*., 1995
**Marseille (France) ?-?**		17 ± 2 (4–36)			1.3 ± 0.2 (0.1–3.7)	9.5 ± 1.2 (1.2–28.3)	Baroli *et al*., 2001
**Ischia (Italy) ?-?**		17 ± 3 (5.0–58.0)			0.7 ± 0.2 (0.1–3.9)	11.2 ± 9.8 (1.8–18.3)	Baroli *et al*., 2001
**Liscia Bay (Sardinia) 1978–1994**		6 ± 3 (3–13)			0.6 ± 0.1 (0.3–0.8)	5.2 ± 8.0 (1.7–36.6)	Baroli *et al*., 2001

Concentrations were expressed in μg g_DW_^-1 ^(mean ± standard deviation, minimum – maximum).

**Table 5:** Mean metal concentrations in different tissues of *P. oceanica*.

	N	Chromium	Nickel	Copper	Zinc	Cadmium	Lead
Leaves	30	1.6 ± 1.5	22.9 ± 10.2	11.1 ± 6.5	109.3 ± 41.1	2.8 ± 0.9	5.2 ± 3.8
Scales	28	19.7 ± 12.0	7.6 ± 5.6	9.5 ± 2.8	20.7 ± 4.4	1.0 ± 0.3	11.4 ± 7.7
M-W U *Z*		-6.504	4.964*	0.498	6.100*	6.100*	-3.750

Leaf and dead sheath concentrations were expressed in μg g_DW_^-1 ^(mean ± standard deviations) and Mann-Whitney U test Z was used for each metal. N corresponds to the number of samples and significantly different samples are marked as * (p < 0.01).

**Table 6:** Leaf metal concentrations and Repeated Mesured ANOVA F for each metal at each time period at Calvi site.

	**N**	**Cr**	**Ni**	**Cu**	**Zn**	**Cd**	**Pb**
1988	5	0.9 ± 0.2	0 ± 0	6.7 ± 2.2 a	88.0 ± 6.8 ab	4.0 ± 1.3	8.6 ± 7.5 a
1993	12	2.4 ± 2.0	20.5 ± 14.6	9.3 ± 2.0 a	120.7 ± 42.0 b	3.2 ± 1.3	7.7 ± 4.5 a
1994	8	0.9 ± 0.9	29.4 ± 3.9	4.4 ± 2.2 a	123.8 ± 47.5 ab	2.6 ± 0.4	4.9 ± 2.1 ab
2003	5	1.1 ± 0.2	18.9 ± 2.6	21.7 ± 4.2 c	65.8 ± 8.4 a	2.3 ± 0.3	2.0 ± 0.8 b
2004	5	1.0 ± 0	22.6 ± 4.6	15.3 ± 2.1 b	102.1 ± 11.9 ab	2.9 ± 0.4	3.0 ± 0.9 ab
							
Log transformed		No	No	No	No	No	Yes
Sum squares		8.254	809.625	928.829	11543.927	8.462	6.521
Mean square		2.063	269.875	232.207	2885.982	2.115	1.630
df		4	4	4	4	4	4
ANOVA *F*		2.391	2.592	47.482*	3.246*	2.175	4.200*

Leaf concentrations were expressed in μg g_DW_^-1 ^(mean ± standard deviation). Where a significant difference was found, a Tukey's HSD post hoc comparison was used to determine where the differences lay and similar samples were marked with the same letter. N corresponds to the number of samples and significantly different samples are marked as * (p < 0.05).

**Table 7:** Variation of mean metal concentrations in the leaves and dead sheaths of *P. oceanica*.

***P. oceanica *organs**	**Sites**	**Metal levels**
Leaves	Calvi	Zn > Ni > Cu > Pb > Cd Cr

Dead sheaths	Calvi	Se Ni > Zn > Cr >As Cu > Pb > Cd
Dead sheaths	Lumio	Se Cr > As > Zn > N i> Cu > Pb > Cd
Dead sheaths	St Florent	Cr > Se > Zn > Ni > Cu As > Pb > Cd
Dead sheaths	Nonza	Cr Ni Se > As > Zn > Cu > Pb > Cd
Dead sheaths	Macinaggio	Se > Cr > Ni As > Zn > Cu > Pb > Cd
Dead sheaths	Bastia	Cr > Zn Ni Se > Pb > Cu As > Cd

Metal concentrations sampled in the six studied meadows (Calvi, Lumio, St Florent, Nonza, Macinaggio and Bastia).

**Table 8:** Time sampling design according to organs and sites.

**Organs**	**studied metals**	**Sites**	**Sampling time**	**studied period**
Leaves	Cr, Ni, Cu, Zn, Cd, and Pb	Calvi	- 1988 (February and May)	1988 – 2004
			- 1993 (March, June, September and December)	
			- 1994 (February, April and June)	
			- 2003 (November)	
			- 2004 (December)	

Sheaths	Cr, Ni, Cu, Zn, As, Se, Cd and Pb	Calvi	- 2001 (November)	1992 – 2002
		Lumio	- 2002 (February)	(dated back)
		Saint-Florent		
		Nonza		
		Macinaggio		
		Bastia		
